# Serum IL-23, IL-10, and TNF-α predict in-hospital mortality in COVID-19 patients

**DOI:** 10.3389/fimmu.2023.1145840

**Published:** 2023-05-22

**Authors:** Shukur Wasman Smail, Esmaeil Babaei, Kawa Amin, Wayel H. Abdulahad

**Affiliations:** ^1^ Department of Biology, College of Science, Salahaddin University, Erbil, Iraq; ^2^ Department of Biology, School of Natural Sciences, University of Tabriz, Tabriz, Iran; ^3^ Department of Pharmacognosy, College of Pharmacy, Hawler Medical University, Erbil, Iraq; ^4^ College of Medicine, University of Sulaimani, Sulaymaniyah, Iraq; ^5^ Department of Medical Science, Respiratory, Allergy and Sleep Research, Uppsala University, Uppsala, Sweden; ^6^ Department of Rheumatology and Clinical Immunology, University Medical Center Groningen, University of Groningen, Groningen, Netherlands; ^7^ Department of Pathology and Medical Biology, University Medical Center Groningen, University of Groningen, Groningen, Netherlands

**Keywords:** COVID-19, interleukins, mortality, SARS-CoV-2, predictors

## Abstract

**Objective:**

The hyperinflammatory response, caused by severe acute respiratory syndrome-2 (SARS-CoV-2), is the most common cause of death in patients with coronavirus disease 2019 (COVID-19). The etiopathogenesis of this illness is not fully understood. Macrophages appear to play a key part in COVID-19’s pathogenic effects. Therefore, this study aims to examine serum inflammatory cytokines associated with the activation state of macrophages in COVID-19 patients and attempt to find accurate predictive markers for disease severity and mortality risk in hospital.

**Methods:**

180 patients with COVID-19 and 90 healthy controls (HCs) participated in this study. Patients were divided into three different subgroups, mild (n=81), severe (n=60), and critical groups (n=39). Serum samples were collected and IL (Interleukin)-10, IL-23, tumor necrosis factor-alpha (TNF-α), interferon-gamma (IFN-γ), IL-17, monocyte chemoattractant protein-1 (MCP-1) and chemokine ligand 3 (CCL3) were determined by ELISA. In parallel, myeloperoxidase (MPO) and C-reactive protein (CRP) were measured using colorimetric and electrochemiluminescence methods, respectively. Data were collected, and their associations with disease progression and mortality were assessed using regression models and receiver operating characteristic (ROC) curves.

**Results:**

Compared to HCs, a significant increase in IL-23, IL-10, TNF-α, IFN-γ and MCP-1, were observed in COVID-19 patients. Serum levels of IL-23, IL-10, and TNF-α were significantly higher in COVID-19 patients with critical cases compared to mild and severe cases, and correlated positively with CRP level. However, non-significant changes were found in serum MPO and CCL3 among the studied groups. Moreover, significant positive association has been observed among increased IL-10, IL-23 and TNF-α in serum of COVID-19 patients. Furthermore, a binary logistic regression model was applied to predict death’s independent factors. Results showed that IL-10 alone or in combination with IL23 and TNF-α are strongly linked with non-survivors in COVID-19 patients. Finally, ROC curve results uncovered that IL-10, IL-23 and TNF-α were excellent predictors for prognosing COVID-19.

**Conclusion:**

The elevations of IL-10, IL-23, and TNF-α levels were seen in severe and critical cases of COVID-19 patients and their elevations were linked to the in-hospital mortality of the disease. A prediction model shows that the determination of these cytokines upon admission is important and should be done on COVID-19 patients as a way of evaluating the prognosis of the disease. COVID-19 Patients with high IL-10, IL-23, and TNF-α on admission are more likely to experience a severe form of the disease; therefore, those patients should be cautionary monitored and treated.

## 1 Introduction

Coronavirus disease 2019 (COVID-19) is an infectious acute respiratory disease caused by a strain of coronavirus known as SARS-CoV-2. The first case diagnosed with this disease was reported in Wuhan, China in early December 2019 and subsequently spread worldwide. Most COVID-19 patients (81%) experience milder symptoms and recover without supportive treatment. About 14% of patients develop severe respiratory distress. Approximately 5% of patients are classified as critical cases due to the development of rapid respiratory failure that requires ventilatory support and specialized treatment in intensive care units. Until May 7, 2022, 516,792,956 people had been infected with SARS-CoV-2, and 6,249,813 people died. Thus, about 1.21% of people died after being infected with this virus (1). The exact molecular mechanisms of disease progression to acute respiratory distress have not been enlightened. Therefore, identifying biomarkers associated with disease status and predicting the severity of the disease in patients with COVID-19 is required.

Along with the decrease in pressure and oxygen saturation, various biological magnitudes have been used to stratify this disease’s severity and prognosis. Among them, in the serum of COVID-19 patients, considerably high amounts of cytokines and chemokines have been found. TNF-α, IL-6, IL-1b, IFNγ, IL-10, IL-2, IL-4, IL-12, and IL-17 are cytokines that tend to be elevated in COVID-19 (2, 3). At the same time, CCL5, CCL2, CCL3, CXCL8, and CXCL9 are chemokines that their elevations were also recorded in some cases of COVID-19 (4). Multiple immunological pathways are activated, resulting in hyperinflammatory reactions and cytokine storms, manifested as acute respiratory distress syndrome with consequent respiratory failure (5–8).

Macrophages are the major players in viral infection, which are supremely located to dictate the innate defense of the airway and appear to be essential in the pathophysiology of COVID-19 (9). Single-cell RNA sequencing analysis of bronchoalveolar fluid taken from participants suffering from mild or severe COVID-19 shows a disturbed balance of lung macrophage numbers during the development of severe COVID-19. This was indicated by a decrease in tissue-resident alveolar macrophages and an increase in inflammatory monocyte-derived macrophages in patients with severe disease (10). Therefore, macrophages might be linked to the heterogeneity of the disease courses in COVID-19, and analyzing serological markers of activated macrophages may aid in predicting disease severity and mortality.

A range of inflammatory factors is produced by macrophages, such as CCL3, IL-10, MCP-1, MPO, and IL-23 (11–14). It has been noticed that bronchial macrophages in the critical COVID-19 subgroup, compared to moderate ones, overexpressed the gene encoding for CCL3 (15). Serum levels of CCL3 were also found to be associated with the severity of COVID-19 (16). Furthermore, significant increases in serum IL-10 were found in severe and critical COVID-19 patients who experienced cytokine storms (16). Moreover, an increase in serum MPO in COVID-19 patients was observed, followed by a decrease to a normal level in recovering patients (17). In addition, patients with more severe COVID-19 had a higher level of IL-23 in their blood than those with mild COVID-19 (18).

Although many serum markers have been previously tested as severity and mortality predictors in COVID-19 patients (19–21), their exact impacts on respiratory failure and disease progression remain challenging. In this study, we attempt to assess serum markers associated with macrophage activation that may predict disease progression and in-hospital mortality in patients with mild, severe, and critical COVID-19.

## 2 Materials and methods

### 2.1 Patients and study samples

This prospective multi-center observational study was carried out from May 1 to October 1, 2021, with participants who attended the emergency departments of the hospitals in the Kurdistan region-Iraq, including four cities (Erbil, Duhok, Sulaymaniyah, and Kirkuk).

270 participants were included in this study. These participants were classified into two groups: COVID-19 positive (n=180) with a mean age of 43.92 ± 0.822 years, and COVID-19 negative (HC; n=90) with a mean age of 41.20 ± 1.354 years (**Table 1**). The COVID-19-positive patients were confirmed based on molecular (RT-PCR), laboratory, and radiological findings that followed the WHO criteria (22); they were further sub-classified into three groups based on clinical manifestations: mild (n=81), severe (n=60), and critical (n=39), as the guideline explains it in China (23). According to this protocol, mild cases either don’t exhibit pneumonia or exhibit mild pneumonia; severe cases manifest pneumonia characterized by shortness of breath and their SpO2< 93; critical patients suffer from respiratory failure, septic shock, or multiple organ failure; they had died during the study (23). All COVID-19 patients were non-vaccinated; the patients did not involve in any therapeutic intervention. Regarding the HCs, they accompanied COVID-19 patients in emergency departments. They were asymptomatic and had negative RT-PCR. After taking blood samples from all the volunteers on admission to the hospital, their serums were preserved at -80 °C until use.

**Table 1:** Clinical and demographic characteristics of HCs and COVID-19 patients.

Parameters	HCs (Mean ± SEM)	Mild (Mean ± SEM)	Severe (Mean ± SEM)	Critical (Mean ± SEM)	p-value
n	90	81	60	39	
Age (years)	41.20 ± 1.354	43.02 ± 1.203	44.27 ± 1.191	52.67 ± 1.160	<0.0001
Gender (m/f)	44/46	45/36	34/26	20/19	
BMI (kg/m^2^)	26.81 ± 0.488	26.79 ± 0.474	30.54 ± 0.776	29.96 ± 0.915	<0.0001
CRP (mg/l)	0.780 ± 0.093	5.200 ± 1.523	67.20 ± 7.417	65.82 ± 4.841	<0.0001
Lymphocyte (10^3/µL)	2.091 ± 0.063	1.320 ± 0.058	1.483 ± 0.057	0.964 ± 0.092	<0.0001
Neutrophil (10^3/µL)	4.229 ± 0.117	8.274 ± 0.426	6.805 ± 0.342	15.08 ± 0.935	<0.0001
NLR	2.123 ± 0.074	7.444 ± 0.543	5.269 ± 0.464	20.02 ± 1.742	<0.0001
IL-10 (pg/ml)	1.465 ± 0.128	2.433 ± 0.177	6.479 ± 0.262	10.87 ± 0.800	<0.0001
IL-23 (pg/ml)	49.37 ± 2.590	107.7 ± 5.567	156.6 ± 10.19	238.7 ± 12.56	<0.0001
TNF-α (pg/ml)	6.312 ± 0.284	11.94 ± 0.916	9.566 ± 0.814	20.66 ± 1.135	<0.0001
IFN-γ (pg/ml)	25.22 ± 0.280	22.62 ± 0.773	21.82 ± 1.140	30.49 ± 1.229	<0.0001
IL-17 (pg/ml)	35.16 ± 1.017	38.49 ± 2.978	46.23 ± 1.468	45.78 ± 2.921	0.0005
MCP-1 (pg/ml)	16.38 ± 0.768	20.25 ± 0.938	22.32 ± 1.291	24.40 ± 0.627	<0.0001
CCL3 (pg/ml)	106.5 ± 3.362	105.2 ± 3.105	109.2 ± 3.752	99.91 ± 3.632	0.474
MPO (U/I)	56.73 ± 3.009	51.09 ± 3.487	59.07 ± 3.012	61.17 ± 2.402	0.176
Co-morbidities					
HTN	0	10	5	7	
DM	0	5	2	4	
ESRD	0	0	1	1	
Multiple Co-morbidities	0	10	16	10	
Obesity	0	13	16	6	
COPD	0	0	1	1	
Others	0	2	1	2	

One-way ANOVA was used for the comparison of parameters. Tukey test was used as post-hoc test for multiple comparisons. BMI, body mass index; CCL3, chemokine ligand 3; COPD, chronic obstructive pulmonary disease, CRP, C-reactive protein; DM, diabetes mellitus; ESRD, end-stage renal disease; f, female; HCs, healthy controls; HTN, hypertension; IFN-γ, interferon-gamma; IL, interleukins, m, male; MCP-1, monocyte chemoattractant protein-1; MPO, myeloperoxidase; NLR, neutrophil-to-lymphocyte ratio; n, number of participants and TNF-α, tumor necrosis factor- alpha. Data were presented as mean ± standard error of the mean (SEM), Probability (p)-value less than 0.05 was considered significant.

The hospital’s data management system obtained demographic data, medical history, some laboratory data (lymphocyte and neutrophil) and comorbidities. The comorbidities considered were multiple comorbidities (31.86%), obesity (30.97%), hypertension (HTN) (19.47%), diabetes mellitus (DM) (9.735%), chronic obstructive pulmonary disease (COPD) (1.770%), end-stage renal disease (ESRD) (1.770%), and other diseases (4.425%).

### 2.2 Measurement of serum parameters

According to the manufacturers ‘ instructions, serum levels of IL-10, IL-23, IL-17, CCL3, MCP-1 were measured by Elabscience^®^ ELISA kits (Elabscience Company, USA), whereas levels of TNF-α and IFN-γ were measured using MyBioSource^®^ ELISA kits (Mybiosource, USA). Immunoturbidimetry has been performed to measure the CRP concentration using Cobas c311 (Cobas, Roche Diagnostic, Mannheim, Germany). According to the manufacturers’ instructions, the serum level of MPO was determined by MPO assay kits (Elabscience Company, USA).

### 2.3 Statistical analysis

All the data fulfilled the criteria of parametric tests since they passed normality tests (Kolmogorov-Smirnov, Shapiro-Wilk, and D’Agostino tests). The chi-square test and one-way ANOVA test were used to analyze demographic and clinical characteristics. The one-way ANOVA test was applied to compare laboratory parameters between groups and Tukey test was used as *post-hoc* test for multiple comparisons. A Pearson correlation was used to explore the correlations between different parameters. Binary logistic regression was applied to find the impact of independent factors in predicting the non-survival probability of COVID-19 patients. The predictive significance of the biomarkers was calculated using ROC analysis as a plot of the sensitivity of the respective test against the false-positive rate for possible cut-off levels. A combination of IL-10 and IL-23 for ROC curved was found via the probability of binary logistic regression in SPSS. The optimum cut-off was found via the Youden index method (24) in MedCalc. The area under the curve (AUC), positive predictive value (PPV), negative predictive value (NPV), and their confidence intervals (CI) were obtained by ROC curve analysis.

For doing statistical analysis, the following programs were utilized: The statistical package for social sciences 27 (SPSS 27, IBM, USA), MedCalc 20 (MedCalc software Ltd., Belgium), and GraphPad 9 (GraphPad Software Inc., USA). A *P*-value of less than 0.05 was considered statistically significant.

## 3 Results

### 3.1 Patients and study samples

In total 270 participants were enrolled in this study, 180 (66.67%) were COVID-19 positive, and 90 (33.33%) were HCs. Among the positive patients, 81 (45%) developed the mild disease, 60 (33.33%) with severe, and 39 (21.67%) were critical cases, based on clinical guidelines in China (23). In the last group, all patients (n=39) died in the hospital during the study.

The demographic characteristics and the associated comorbidities and the comparison of these variables among the groups of COVID-19 patients and HCs are shown in **Table 1**. The mean age of HCs was 41.20 ± 1.354 years; 44 (48.89%) were male, while 46 (51.11%) of them were female. The mean age of COVID-positive patients was 43.92 ± 0.822 years, 99 (55%) were male, and 81 (45%) were female. There was a non-significant difference in the age between HCs and COVID-19 (p-value=0.072).

In comparison to HCs, a significant difference (p-value<0.0001) was observed in the age of critical groups of COVID-19 patients. There was no significant difference in the mean age of mild and severe COVID-19 patients compared to HCs.

Significant differences were recorded for BMI between HCs (26.81 ± 0.488) with each of the severe and critical groups of COVID-19 patients (mean of BMI=30.54 ± 0.776 and 29.96 ± 0.915 for severe and critical groups, respectively) with a p-value<0.0001. There was a non-significant difference between mild (26.79 ± 0.474) and HCs (26.81 ± 0.488).

Regarding CRP, non-significant differences were documented between HC and mild groups (p-value=0.777); Severe and critical groups (p-value=0.996). On the other hand, between other groups, there were considerable differences with their p-value<0.0001. In terms of hematological parameters, all COVID-19 groups exhibited a significant increase in lymphocyte and neutrophil counts (p-value < 0.0001) with a significant decrease in the neutrophil-to-lymphocyte ratio (NLR) when compared to the HCs (**Table 1**).

### 3.2 Elevated levels of IL-10, IL-23, TNF-α and IFN-γ in serum samples of COVID-19 patients with critical cases compared to mild and severe cases

**Figure 1:**
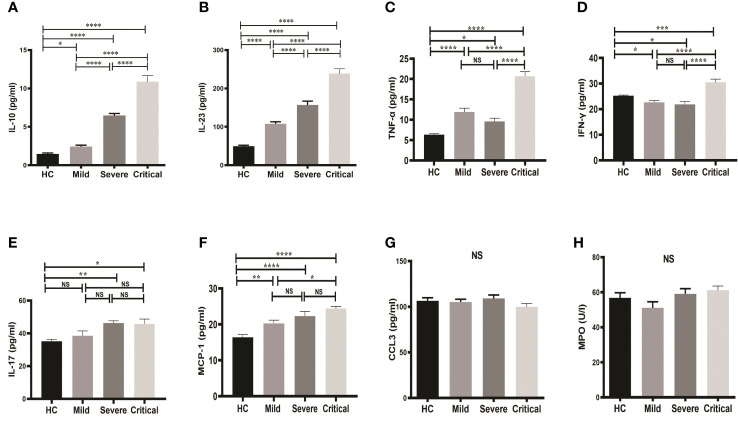
Comparison of serum markers on admission between groups of COVID-19 patients and HCs. One-way ANOVA was used for the comparison of **(A)** IL-10; **(B)** IL-23; **(C)** TNF-α; **(D)** IFN-γ; **(E)** IL-17; **(F)** MCP-1; **(G)** CCL3; and **(H)** MPO. Tukey test was used as *post-hoc* test for multiple comparisons. CCL3, chemokine ligand 3; CRP, C-reactive protein; HC, healthy control; IFN-γ, interferon-gamma; IL-10, interleukin-10; IL-23, interleukin-23; IL-17, interleukin-17; MCP-1, monocyte chemoattractant protein-1; MPO, myeloperoxidase and TNF-α, tumor necrosis factor-alpha. Probability (p)-value less than 0.05 was considered significant. *p-value < 0.05,**p-value < 0.01, ***p-value < 0.001 and ****p-value < 0.0001; ns, not significant.

Serum concentrations of IL-10, IL-23, TNF-α, IFN-γ, IL-17, CCL3, MCP-1 and MPO of patients and HCs were measured. Serum levels of IL-10 showed a significant increase (p-value <0.0001) in both critical (10.87 ± 0.800) and severe groups (6.479 ± 0.262) compared to HCs (**Figure 1A**). On the other hand, there was also an observable difference in IL-10 between the mild group (2.433 ± 0.177) and HCs (1.465 ± 0.128) (**Figure 1A**). Levels of IL-10 were significantly higher in critical cases compared to both severe and mild cases.

Similar results were found for serum IL-23 (**Figure 1B**). Serum IL-23 was significantly higher in all groups of COVID-19 patients compared to HCs. As shown in **Figure 1B**, serum levels of IL-23 were markedly higher (p-value < 0.0001) in the critical (238.7 ± 12.56) and severe groups (156.6 ± 10.19) compared to the mild group (107.7 ± 5.567).

Furthermore, serum levels of TNF-α, IFN-γ were significantly elevated in COVID-19 patients with critical case compared to mild and severe stage (**Figures 1C, D**). TNF-α was significantly higher in all patients’ groups in comparison to HCs, whereas IFN-γ was found to be significantly decreased in the mild and severe COVID-19 groups, but significantly increased in the critical COVID-19 group compared to the HCs.

IL-17 level was also increased in COVID-19 patients with severe and critical stages compared to HCs, however, no significant differences were observed among the 3 patient groups (**Figure 1E**). Similarly, serum level of MCP-1 was significantly elevated in all patient groups compared to HCs, whereas no differences were observed among patient groups except a slight increase in patients with critical case compared to mild stage (**Figure 1F**). Moreover, no changes in serum levels of both CCL3 and MPO were detected; neither between groups of COVID-19 patients and HCs nor between the patient groups (**Figures 1G, H**).

### 3.3 Significant positive interplay among increased IL-10, IL-23, and TNF-α in serum of COVID-19 patients

**Figure 2:**
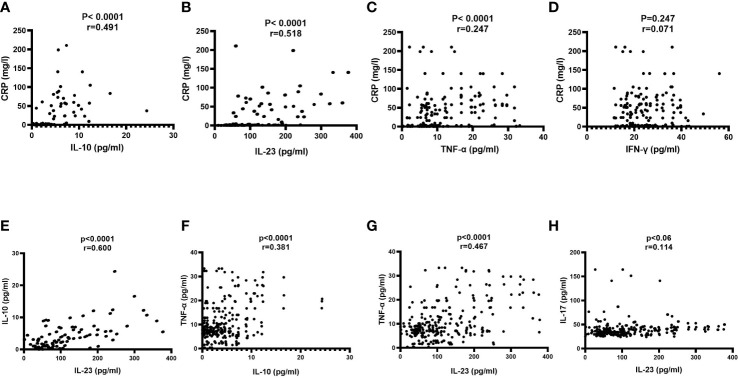
Correlation of serum markers on admission with each other. A Pearson correlation test was used for the association between **(A)** CRP and IL-10; **(B)** CRP and IL-23; **(C)** CRP and TNF-α; **(D)** CRP and IFN-γ; **(E)** IL-10 and IL-23; **(F)** IL-10 and TNF-α **(G)** TNF-α and IL-23 and **(H)** IL-17 and IL-23. CRP, C-reactive protein; IFN-γ, interferon-gamma; IL-10, interleukin-10; IL-23, interleukin-23; IL-17, interleukin-17 and TNF-α, tumor necrosis factor-alpha. Probability (p)-value less than 0.05 was considered significant, and the correlation coefficient (r) shows the degree of correlation.

To gain insight into the key immunological responses and inflammation, we correlated the levels of IL-10, IL-23, TNF-α, and IFN-γ with CRP (**Figures 2A–D**). It can clearly seen that IL-10, IL-23 and TNF-α showed the strong significant positive correlations with CRP, and the degree of their associations were (r=0.491, p-value<0.0001), (r=0.518, p-value<0.0001), and (r=0.247, p-value<0.0001), respectively (**Figures 2A–C**). In contrast, IFN-γ witnessed a non-significant correlation with CRP (r=0.071, p-value=0.247) (**Figures 2D**).

Next, we assessed the mutual relationships among IL-10, IL-23 and TNF-α. As shown in **Figures 2E–G**, significant positive association has been noticed between IL-10 and IL-23 (r=0.600, p-value<0.0001); IL-10 and TNF-α (r=0.381, p-value<0.0001), and IL-23 and TNF-α (r=0.467, p-value<0.0001). With regards to the association of IL-23 in polarizing Th17 and the release of IL-17, we observed non-significant correlation between IL-23 and IL-17 (r=0.114, p-value=0.06) (**Figure 2H**).

### 3.4 IL-10, IL-23 and TNF-α as putative markers for predicting mortality in COVID-19 illness

**Figure 3:**
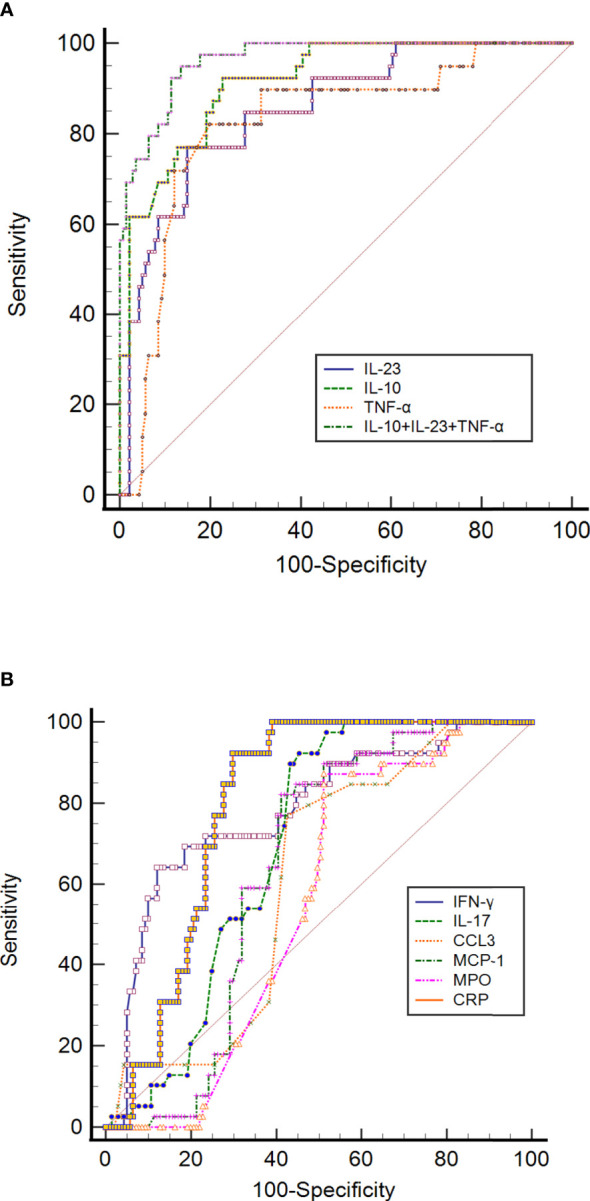
ROC curve analysis of **(A)** CRP, IL-10, IL-23, TNF-α, and IL-10+IL-23+TNF-α, and **(B)** IFN-γ, IL-17, CCL3, MCP-1, MPO and CRP to predict mortality in SARS-CoV-2 infection. The ROC curve figure was created by MedCalc20. The COVID-19 patients were divided into survived and non-survived groups considering as status in the ROC curve analysis. CCL3, chemokine ligand 3; CRP, C-reactive protein; IFN-γ, interferon-gamma; IL-10, interleukin-10; IL-23, interleukin-23; IL-17, interleukin-17; MCP-1, monocyte chemoattractant protein-1; MPO, myeloperoxidase; ROC, receiver operating characteristic curve and TNF-α, tumor necrosis factor-alpha.

To assess the prognostic usefulness of laboratory markers for predicting mortality in patients with potential COVID-19, ROC curves were drawn for 180 patients with COVID-19. The significance of the IL-23 concentration in terms of detecting COVID-19 mortality is 76.92% sensitivity and 85.11% specificity. The cut-off value from which mortality can be assumed is 191.4, and the AUC is 0.855. The calculation of the NPV turned out to be 93%. The PPV is 58.8% (**Table 2**; **Figure 3A**).

**Table 2:** ROC curve analysis of serum laboratory markers to predict mortality of SARS-CoV-2 infection.

Variables	AUC	95% CI	p-value	Cut-off	Sensitivity	95% CI	Specificity	95% CI	PPV	95% CI	NPV	95% CI
IL-23	0.855	0.795 - 0.903	<0.0001	>191.4	76.92	60.7 - 88.9	85.11	78.1 - 90.5	58.8	48.2 - 68.7	93.0	88.2 - 96.0
IL-10	0.916	0.866- 0.952	<0.0001	>6.07	92.31	79.1 - 98.4	77.30	69.5 - 83.9	52.9	45.0 - 60.7	97.3	92.4 - 99.1
TNF-α	0.824	0.761 - 0.877	<0.0001	>15.43	82.05	66.5 - 92.5	80.14	72.6 - 86.4	53.3	44.3 - 62.2	94.2	89.1 - 96.9
IL-10+IL-23+ TNF-α	0.965	0.926 - 0.986	<0.0001	>0.132	94.87	82.7 - 99.4	86.52	79.8 - 91.7	66.1	56.0 - 74.9	98.4	94.0 - 99.6
IFN-γ	0.781	0.713 - 0.839	<0.0001	>29.52	64.10	47.2 - 78.8	87.94	81.4 - 92.8	59.5	47.0 - 70.9	89.9	85.3 - 93.1
IL-17	0.693	0.620 - 0.759	<0.0001	>37.72	92.31	79.1 - 98.4	54.61	46.0 - 63.0	36.0	31.5 - 40.8	96.2	89.5 - 98.7
CCL3	0.608	0.533 - 0.680	0.016	≤95.56	76.92	60.7 - 88.9	57.45	48.8 - 65.7	33.3	27.9 - 39.3	90.0	83.3 - 94.2
MCP-1	0.636	0.562 - 0.707	0.0007	>22.56	82.05	66.5 - 92.5	58.87	50.3 - 67.1	35.6	30.1 - 41.4	92.2	85.7 - 95.9
MPO	0.551	0.475 - 0.625	0.229	>43.93	87.18	72.6 - 95.7	48.94	40.4 - 57.5	32.1	27.9 - 36.6	93.2	85.7 - 97.0
CRP	0.798	0.731-0.854	<0.0001	>33.68	92.31	79.1 - 98.4	70.21	61.9 - 77.6	46.2	39.6 - 52.9	97.1	91.7 - 99.0

ROC curve analysis was done by MedCalc 20. The COVID-19 patients were divided into survived and non-survived considering as status in the ROC curve analysis. AUC, area under curve; CCL3, chemokine ligand 3; CI, confidence interval; CRP, C-reactive protein; IFN-γ, interferon-gamma; IL-10, interleukin-10; IL-23, interleukin-23; IL-17, interleukin-17; MCP-1, monocyte chemoattractant protein-1; MPO, myeloperoxidase; NPV, negative predictive value; PPV, positive predictive value; ROC, receiver operating characteristic curve and TNF-α, tumor necrosis factor- alpha. Probability (p)-value less than 0.05 was considered significant.

The ROC curve of IL-10 for prognosing COVID-19 was also analyzed. Its results revealed that the AUC of IL-10 for predicting mortality was 0.916 (95% CI 0.866-0.952), with a cut-off value of 6.07. It had a sensitivity of 92.31% and a specificity of 77.30%, with NPV and PPV of 97.3% and 52.9%, respectively (**Table 2**; **Figure 3A**). AUCs and cut-off value were also found for TNF-α, with values of 0.824 and 15.43, respectively (**Table 2**; **Figure 3A**). The result also showed that the AUCs with the best performance in predicting mortality were IL-10, IL-23, and TNF-α. The combination of IL-10, IL-23 and TNF-α achieves much higher AUC values (0.965) than the other inflammatory markers concerning mortality prediction. The NPV and PPV of this combination were 98.4% and 66.1%, respectively. The details of the ROC curve characteristics for other measured proteins indicates their useless values in predicting mortality as shown in **Table 2**; **Figure 3B**.

Next, multivariate binary logistic regression was performed to predict mortality risk in COVID-19 patients. After adjusting for age, IL-10, IL-23, TNF-α, IFN-γ, IL-17, CCL3, MCP-1 and MPO was used in the model as prognostic factors to predict mortality and death, and to discriminate the non-survivor patients from the survivors. Forward conditional analysis showed that three incremental degrees of adjustment in multivariate binary logistic regression models were significantly associated with death. We documented that IL-10 (p-value<0.0001, OR= 1.813) in model 1; IL-10 (p-value<0.0001, OR= 1.887), and TNF-α (p-value<0.0001, OR= 1.173) in model 2 and IL-10 (p-value<0.0001, OR= 1.836), IL-23 (p-value=0.01, OR= 1.002) and TNF-α (p-value<0.0001, OR= 1.173) in model 3 were significantly linked with mortality in COVID-19 patients. The lack of improvement in explanatory power by other parameters such as MPO, IFN-γ, IL-17, CCL3, MCP-1, and CRP led to their exclusion from the forward conditional analysis, as shown in **Table 3**.

**Table 3:** Binary logistic regression of serum markers to predict COVID-19 mortality (Adjusting for age).

Variables		B	p-value	OR	95% CI for OR	
Model 1	IL-10	0.595	<0.0001	1.813	1.501	2.189
Model 2	IL-10	0.635	<0.0001	1.887	1.505	2.367
	TNF-α	0.159	<0.0001	1.173	1.094	1.258
Model 3	IL-10	0.607	<0.0001	1.836	1.438	2.343
	IL-23	0.009	0.01	1.009	1.002	1.017
	TNF-α	0.159	<0.0001	1.173	1.079	1.275

Binary logistic regression was done by SPSS 27. The COVID-19 patients were divided into survived and non-survived considered as dependent variables (dichotomous variables), it was adjusted for age. In forward conditional binary logistic regression, model 1 (IL-10) and model 2 (IL-10 and TNF-α) and model 3 (IL-10, IL-23 and TNF-α) were considered predictors for mortality in COVID-19. B, regression coefficient; CI, confidence interval; IL-10, interleukin-10; IL-23, interleukin-23; OR, odd ratio and TNF-α, tumor necrosis factor- alpha. Probability (p)-value less than 0.05 was considered significant.

## 4 Discussion

Excessive pulmonary macrophages and cytokine storm are dominant in COVID-19 patients (25) and may contribute to lung fibrosis and tissue damage. It is believed that SARS-CoV-2 bind to the ACE2 receptor on macrophages (M1 subtype) and induces the release of inflammatory factors including IL-23, CCL3, and MPO (11, 12, 14). On the other hand, M2 (a subtype of macrophage) releases IL10 as a negative feedback mechanism to control inflammation (26). A dysregulated balance of M1 and M2 macrophage subtypes in the lungs of COVID-19 patients has been confirmed during the progression of severe COVID-19. Therefore, assessment of serological markers associated with activated macrophages may aid in predicting disease severity and mortality. In this study, we demonstrated an increase in IL-10, IL-23 and TNF-α in serum samples of COVID-19 patients during mild disease and continue to increase in the severe stage and reach the highest level in the critical phase of the disease. Importantly, the combination of serum levels of IL-10, IL-23 and TNF-α were strongly linked with non-survivors in COVID-19 patients. Therefore, monitoring changes in the serum levels of IL-10, IL-23, and TNF-α may help in the early identification and prediction of COVID-19 disease progression.

The higher serum concentration of IL-10 in the severe and critical group of COVID-19 patients has been noticed in this study. Increased serum levels of IL-10 has been previously demonstrated as a prognostic factor in COVID-19 patients (16, 27, 28). This increase in IL-10 remains elusive since IL-10 is a classical anti-inflammatory cytokine. Elevation in IL-10 in COVID-19 patients can be justified as an effort to moderate hyper-inflammation and prevent tissue damage, but it fails to suppress the inflammation. It is highly unlikely that increased IL-10 expression is attributed to regulatory T cells (Treg), as these Treg cells were shown to be decreased in moderate and severe cases of COVID-19 (29). Therefore, the elevated level of serum IL-10 in critical cases of COVID-19 may be related to activated monocytes and macrophages, which are the primary producers of this cytokine (30). On the other hand, IL-10 may have pro-inflammatory properties. Indeed, high-dose IL-10 treatment in patients with endotoxemia or Crohn’s disease was associated with undesired pro-inflammatory effects by enhancing the production of other pro-inflammatory cytokines, such as IFN-γ (31). It was shown that IL-10 potentiated the lipopolysaccharide (LPS) and encouraged a rise in IFN-γ. Thus, it is possible that this also occurs in patients with COVID-19, in which the combination of high-dose IL-10 and bacterial products drives an inflammatory cascade in COVID-19 patients. To support this hypothesis, our results confirmed that the concentration of IL-10 was directly associated with both CRP and the pro-inflammatory cytokines IL-23 and TNF-α in COVID-19 patients. It seems that IL-10 is involved in the up-regulation of inflammatory reactions in COVID-19 and might be implicated in a cytokine storm. We have shown that the serum level of IL-10 has a good predictive value of disease progression in COVID-19. In line with our findings, other researchers also considered IL-10 as a prognostic factor for expecting the severity of COVID-19 (28, 32).

In addition to elevated IL-10 levels, TNF-α and IL-23 were also increased in serum of COVID-19 patients and correlated positively with CRP levels. It has been previously investigated that CRP can induce TNF-α and IL-23 production by monocytes (33, 34), so the elevation of TNF-α and IL-23 in COVID-19 patients might be related to CRP-induced monocytes. IL-23, in turn, can promote Th17 proliferation and differentiation, which releases the pro-inflammatory cytokine IL-17 and enhances inflammation (35). However, no significant correlation was observed between the IL-23 and IL-17 levels in our patients cohort. Regarding TNF-α, it has been well established that it is a risk factor for death in COVID-19 patients with severe or critical disease (36).

In our study, we observed a significant positive correlation between increased serum levels of IL-10, IL-23 and TNF-α in COVID-19 patients. This suggests that these three cytokines may be produced from a single cell source, such as monocytes, which are known to release all these cytokines when activated. Interestingly, we found that IL-10, IL-23, and TNF-α were strongly associated with COVID-19 mortality, as demonstrated by ROC curve and multivariate regression analysis. Hospitalized patients with higher serum concentrations of IL-10, IL-23, and TNF-α had a noticeably higher risk of death. Therefore, assessment of serum levels of these three cytokines may serve as an early indicator of disease progression and may help make decisions related to treatment to prevent disease complications and death.

This study is not free from limitations. First, a small sample size is one of the limitations that might increase the standard error of variables. Second, blood was only drawn from patients at the time of admission. The result would have been more meaningful if the blood had been retaken at regular intervals and the biomarkers re-assessed. Third, the time elapsed between the onset of symptoms and the taking of the sample in the emergency department was not considered since this information was not available for most COVID-19 patients. Fourth, only 39 critical COVID-19 patients (deaths) were recruited in this study, and the causes of death were not corroborated by autopsy in any of the cases. Fifth, the lack of monocyte count, which plays an important role in the immune response to infections, may be seen as another limitation of our study. Last, multisystem inflammatory syndrome (MAS) in COVID-19 patients was not evaluated. This is because the diagnosis of MAS requires strict criteria that involve a combination of laboratory, radiological, and clinical findings. As a result, the findings of this study may not be generalizable to patients with COVID-19 who develop MAS.

## 5 Conclusion

Measurement of IL-10, IL-23, and TNF-α are the best markers for predicting in-hospital mortality in COVID-19; they have potential prognostic values in SARS-CoV-2 infection. In addition, determination of these cytokines in COVID-19 patients at hospital admission may help save their lives because they are bad prognostic variables. Those three cytokine levels may be used to predict whether patients are at risk for respiratory failure, organ damage, and death. Early in the illness, a prediction model based on cytokine levels might help guide healthcare allocation and priority for those most at risk. The predictive usefulness of these cytokines may also aid therapeutic intervention. Future studies with larger sample sizes must be warranted to confirm the finding of our cross-sectional study. Dynamic analysis of IL-10, IL-23, and TNF-α should be done through consecutive samples taken at several time points to get insights into their kinetics.

## Data availability statement

The original contributions presented in the study are included in the article/supplementary material. Further inquiries can be directed to the corresponding author.

## Ethics statement

The studies involving human participants were reviewed and approved by the Ethics Committee of Salahaddin University-Erbil (SUE) approved this study (approval number: R03-021; 92 approved on April 27, 2021), and all participants gave their written informed consent before blood withdrawal. For patients who were unable to sign due to life-threatening emergencies, we asked their relatives, who had substitute decision-makers, legal guardians, or power of attorney for the patient. The patients/participants provided their written informed consent to participate in this study.

## Author contributions

All authors contributed equally to this manuscript. Methodology and data analysis were performed by SS. The manuscript draft was written by KA. The conception of this study was supervised by WA. All authors commented on previous versions of the manuscript. All authors contributed to the article and approved the submitted version.

## References

[1] Hopkins J. COVID-19 dashboard by the center for systems science and engineering (CSSE) at johns Hopkins university (JHU) (2022). Available at: https://coronavirus.jhu.edu/map.html. 10.1016/S1473-3099(22)00434-0 PMC9432867 36057267

[2] Liu Y Zhang C Huang F Yang Y Wang F Yuan J. Elevated plasma levels of selective cytokines in COVID-19 patients reflect viral load and lung injury. Natl Sci Rev (2020) 7(6):1003–11. doi: 10.1093/nsr/nwaa037 PMC7107806 34676126

[3] Hu H Pan H Li R He K Zhang H Liu L. Increased circulating cytokines have a role in COVID-19 severity and death with a more pronounced effect in males: a systematic review and meta-analysis. Front Pharmacol (2022) 13:802228. doi: 10.3389/fphar.2022.802228 35237162 PMC8883392

[4] Coperchini F Chiovato L Ricci G Croce L Magri F Rotondi M. The cytokine storm in COVID-19: further advances in our understanding the role of specific chemokines involved. Cytokine Growth factor Rev (2021) 58:82–91. doi: 10.1016/j.cytogfr.2020.12.005 33573850 PMC7837329

[5] Meftahi GH Jangravi Z Sahraei H Bahari Z. The possible pathophysiology mechanism of cytokine storm in elderly adults with COVID-19 infection: the contribution of “inflame-aging”. Inflammation Res (2020) 69(9):825–39. doi: 10.1007/s00011-020-01372-8 PMC7289226 32529477

[6] Bahari Z Jangravi Z Ghoshooni H Afarinesh MR Meftahi GH. Pharmacological mechanism of immunomodulatory agents for the treatment of severe cases of COVID-19 infection. Inflammation Res (2021) 70(4):389–405. doi: 10.1007/s00011-021-01445-2 PMC7894237 33608746

[7] Gupta A Marzook H Ahmad F. Comorbidities and clinical complications associated with SARS-CoV-2 infection: an overview. Clin Exp Med (2022) 1–19. doi: 10.1007/s10238-022-00821-4 PMC8972750 35362771

[8] Smail SW Saeed M Twana A Khudhur ZO Younus DA Rajab MF. Inflammation, immunity and potential target therapy of SARS-COV-2: a total scale analysis review. Food Chem Toxicol (2021) 150:112087. doi: 10.1016/j.fct.2021.112087 33640537 PMC7905385

[9] Byrne AJ Mathie SA Gregory LG Lloyd CM. Pulmonary macrophages: key players in the innate defence of the airways. Thorax (2015) 70(12):1189–96. doi: 10.1136/thoraxjnl-2015-207020 26286722

[10] Liao M Liu Y Yuan J Wen Y Xu G Zhao J. Single-cell landscape of bronchoalveolar immune cells in patients with COVID-19. Nat Med (2020) 26(6):842–4. doi: 10.1038/s41591-020-0901-9 32398875

[11] Verreck FAW Td B Langenberg DML MA H Kramer M Vaisberg E. Human IL-23-producing type 1 macrophages promote but IL-10-producing type 2 macrophages subvert immunity to (myco)bacteria. Proc Natl Acad Sci (2004) 101(13):4560–5. doi: 10.1073/pnas.0400983101 PMC384786 15070757

[12] Bhavsar I Miller CS Al-Sabbagh M. Macrophage inflammatory protein-1 alpha (MIP-1 alpha)/CCL3: as a biomarker. Gen Methods Biomark Res Applications (2015) 223–49. doi: 10.1007/978-94-007-7696-8_27

[13] Sanin DE Prendergast CT Mountford AP. IL-10 production in macrophages is regulated by a TLR-driven CREB-mediated mechanism that is linked to genes involved in cell metabolism. J Immunol (2015) 195(3):1218–32. doi: 10.4049/jimmunol.1500146 PMC4505959 26116503

[14] Shaeib F Khan SN Thakur M Kohan-Ghadr H-R Drewlo S Saed GM. The impact of myeloperoxidase and activated macrophages on metaphase II mouse oocyte quality. PloS One (2016) 11(3):e0151160–e. doi: 10.1371/journal.pone.0151160 PMC4794194 26982351

[15] Chua RL Lukassen S Trump S Hennig BP Wendisch D Pott F. COVID-19 severity correlates with airway epithelium–immune cell interactions identified by single-cell analysis. Nat Biotechnol (2020) 38(8):970–9. doi: 10.1038/s41587-020-0602-4 32591762

[16] Chi Y Ge Y Wu B Zhang W Wu T Wen T. Serum cytokine and chemokine profile in relation to the severity of coronavirus disease 2019 in China. J Infect Dis (2020) 222(5):746–54. doi: 10.1093/infdis/jiaa363 PMC7337752 32563194

[17] Shrivastava S Chelluboina S Jedge P Doke P Palkar S Mishra AC. Elevated levels of neutrophil activated proteins, alpha-defensins (DEFA1), calprotectin (S100A8/A9) and myeloperoxidase (MPO) are associated with disease severity in COVID-19 patients. Front Cell Infect Microbiol (2021) 1056:751232. doi: 10.3389/fcimb.2021.751232 PMC8566808 34746027

[18] Markovic SS Jovanovic M Gajovic N Jurisevic M Arsenijevic N Jovanovic M. IL 33 correlates with COVID-19 severity, radiographic and clinical finding. Front Med (Lausanne) (2021) 8:749569. doi: 10.3389/fmed.2021.749569 34917631 PMC8669591

[19] Suranadi IW Sucandra IMAK Fatmawati NND Wisnawa ADF. A retrospective analysis of the bacterial infections, antibiotic use, and mortality predictors of COVID-19 patients. Int J Gen Med (2022) 15:3591. doi: 10.2147/IJGM.S351180 35392031 PMC8983054

[20] Xu Y Yang H Wang J Li X Xue C Niu C. Serum albumin levels are a predictor of COVID-19 patient prognosis: evidence from a single cohort in chongqing, China. Int J Gen Med (2021) 14:2785. doi: 10.2147/IJGM.S312521 34194238 PMC8238547

[21] Zhan L Liu Y Cheng Y Guo W Yang J. Predictive value of Neutrophil/Lymphocyte ratio (NLR) on cardiovascular events in patients with COVID-19. Int J Gen Med (2021) 14:3899. doi: 10.2147/IJGM.S317380 34335053 PMC8318887

[22] World Health Organization. Clinical management of severe acute respiratory infection when middle East respiratory syndrome coronavirus (MERS-CoV) infection is suspected: interim guidance. World Health Org (2019). Available at: https://apps.who.int/iris/handle/10665/178529.

[23] Guo J Wang S Xia H Shi D Chen Y Zheng S. Cytokine signature associated with disease severity in COVID-19. Front Immunol (2021) 12:681516. doi: 10.3389/fimmu.2021.681516 34489933 PMC8418386

[24] Youden WJ. Index for rating diagnostic tests. Cancer (1950) 3(1):32–5. doi: 10.1002/1097-0142(1950)3:1<32::aid-cncr2820030106>3.0.co;2-3 15405679

[25] Criado PR Abdalla BMZ de Assis IC van Blarcum de Graaff Mello C Caputo GC Vieira IC. Are the cutaneous manifestations during or due to SARS-CoV-2 infection/COVID-19 frequent or not? revision of possible pathophysiologic mechanisms. Inflammation Res (2020) 69(8):745–56. doi: 10.1007/s00011-020-01370-w PMC7266387 32488318

[26] Atri C Guerfali FZ Laouini D. Role of human macrophage polarization in inflammation during infectious diseases. Int J Mol Sci (2018) 19(6):1801. doi: 10.3390/ijms19061801 29921749 PMC6032107

[27] Chen G Wu D Guo W. Clinical and immunologic features in severe and moderate forms of coronavirus disease 2019. J Clin Invest (2020) 130(5):2620–9. doi: 10.1172/JCI137244 PMC7190990 32217835

[28] Han H Ma Q Li C Liu R Zhao L Wang W. Profiling serum cytokines in COVID-19 patients reveals IL-6 and IL-10 are disease severity predictors. Emerg Microbes Infect (2020) 9(1):1123–30. doi: 10.1080/22221751.2020.1770129 PMC7473317 32475230

[29] Wang H Wang Z Cao W Wu Q Yuan Y Zhang X. Regulatory T cells in COVID-19. Aging Dis (2021) 12(7):1545–53. doi: 10.14336/ad.2021.0709 PMC8460308 34631206

[30] Morhardt TL Hayashi A Ochi T Quirós M Kitamoto S Nagao-Kitamoto H. IL-10 produced by macrophages regulates epithelial integrity in the small intestine. Sci Rep (2019) 9(1):1223. doi: 10.1038/s41598-018-38125-x 30718924 PMC6362270

[31] Lauw FN Pajkrt D Hack CE Kurimoto M van Deventer SJ van der Poll T. Proinflammatory effects of IL-10 during human endotoxemia. J Immunol (2000) 165(5):2783–9. doi: 10.4049/jimmunol.165.5.2783 10946310

[32] Dhar SK KV Damodar S Gujar S Das M. IL-6 and IL-10 as predictors of disease severity in COVID-19 patients: results from meta-analysis and regression. Heliyon (2021) 7(2):e06155–e. doi: 10.1016/j.heliyon.2021.e06155 PMC7846230 33553782

[33] Geyer CE Newling M Sritharan L Griffith GR Chen H-J Baeten DLP. C-reactive protein controls IL-23 production by human monocytes. Int J Mol Sci (2021) 22(21):11638. doi: 10.3390/ijms222111638 34769069 PMC8583945

[34] Ballou SP Lozanski G. Induction of inflammatory cytokine release from cultured human monocytes by c-reactive protein. Cytokine (1992) 4(5):361–8. doi: 10.1016/1043-4666(92)90079-7 1420997

[35] Boniface K Blom B Liu Y-J de Waal Malefyt R. From interleukin-23 to T-helper 17 cells: human T-helper cell differentiation revisited. Immunol Rev (2008) 226:132–46. doi: 10.1111/j.1600-065X.2008.00714.x PMC3660846 19161421

[36] Jia F Wang G Xu J Long J Deng F Jiang W. Role of tumor necrosis factor-α in the mortality of hospitalized patients with severe and critical COVID-19 pneumonia. Aging (Albany NY) (2021) 13(21):23895–912. doi: 10.18632/aging.203663 PMC8610114 34725309

